# Sleeve Gastrectomy Reverses Hepatic and Circulating Transcriptomic Dysregulation by Restoring Lipid and Energy Metabolism in Diet-Induced Metabolic Dysfunction-Associated Steatotic Liver Disease Rats

**DOI:** 10.1159/mpp/adpag015

**Published:** 2026-09-02

**Authors:** Mohammad Jamal, Wayel Jasem, Mohamed Abu-Farha, Md Zubbair Malik, Salman Al-Sabah, Suleiman Al-Sabah, Carol Dsouza, Fatemah AlOtaibi, Hamad Ali, Yun Ma, Fahd Al-Mulla, Jehad Abubaker

**Affiliations:** a Department of Surgery, College of Medicine, Kuwait University, Kuwait City, Kuwait; b Liver Transplant Service, King’s College Hospital, London, UK; c Roger Williams Institute of Liver Studies at King’s College Hospital, London, UK; d Department of Biochemistry and Molecular Biology, Dasman Diabetes Institute, Kuwait City, Kuwait; e Transational Research Department, Dasman Diabetes Institute, Kuwait City, Kuwait; f Department of Pharmacology and Toxicology, College of Medicine, Kuwait University, Kuwait City, Kuwait; g Department of Medical Laboratory Sciences, College of Allied Health, Kuwait University, Kuwait City, Kuwait; h Department of Genetics and Bioinformatics, Dasman Diabetes Institute, Kuwait City, Kuwait

**Keywords:** Diet-induced metabolic dysfunction-associated steatotic liver disease, Sleeve gastrectomy, Hepatic and lipid metabolism

## Abstract

**Objectives:**

This study aimed to identify key hepatic genes involved in metabolic dysfunction-associated steatotic liver disease (MASLD) pathogenesis and determine whether sleeve gastrectomy (SG) attenuates cafeteria diet-induced hepatic steatosis in a rat model.

**Subject and Methods:**

Thirty-two male Sprague-Dawley rats were divided into four groups: control (lean), diet-induced obesity (DIO)-obese control, DIO-sham, and DIO-SG. Hepatic expression profiling of MASLD-associated genes was performed using RT^2^ Profiler polymerase chain reaction arrays. Circulating adipokines (leptin and adiponectin) and hepatic triglyceride (TG) content were also quantified.

**Results:**

SG significantly reversed obesity-induced upregulation of key lipogenic (*Fasn*, *Srebf1*, *Dgat2*), gluconeogenic (*G6pc*, *Gck*), and inflammatory (*Nfkb1*, *Il1b*) genes, while enhancing fatty acid oxidation and mitochondrial pathways (*Cpt2*, *Ppargc1a*, *Acadl*). Pathway analysis demonstrated significant modulation of peroxisome proliferator-activated receptor and adenosine monophosphate-activated protein kinase signaling, fatty acid oxidation, and insulin sensitivity networks. Circulating leptin and hepatic TG content were significantly reduced following SG, consistent with systemic metabolic improvement. The normalization of *Srebf1* and *Dgat2* highlights restoration of hepatic lipid homeostasis and improved insulin sensitivity.

**Conclusion:**

SG reduced hepatic fat accumulation and mitigated MASLD through broad transcriptional and metabolic reprogramming of lipid, oxidative, and inflammatory pathways. The identification of shared hepatic-whole-blood signatures together with improvements in circulating biomarkers underscores the systemic impact of SG and suggests potential translational targets for noninvasive monitoring and therapeutic intervention.

**Highlights of the Study**

- Obesity-associated fatty liver disease is improved after sleeve gastrectomy.
- Sleeve gastrectomy induces coordinated transcriptional reprogramming of lipid, glucose, and energy metabolism pathways in both liver tissue and whole blood.
- Surgery suppresses key lipogenic and gluconeogenic gene programs while enhancing genes involved in fatty acid oxidation, mitochondrial function, and insulin signaling.

## Introduction

Obesity has become a global epidemic, profoundly affecting health, quality of life, and healthcare systems worldwide. It underpins a broad spectrum of chronic diseases, including type 2 diabetes, metabolic dysfunction-associated steatotic liver disease (MASLD), cardiovascular disease, musculoskeletal disorders, and several obesity-related malignancies [1–4]. Despite continuous efforts, achieving durable and effective weight control remains a major clinical challenge. Metabolic and bariatric surgery (MBS) of which sleeve gastrectomy (SG) is currently the most frequently performed procedure worldwide produces substantial and durable weight loss together with marked improvements in glycemic control, dyslipidemia, and hepatic steatosis, and is now recognized as one of the most effective long-term treatments for obesity and its metabolic complications [5]. A large body of longitudinal data demonstrates that MBS significantly reduces major adverse liver outcomes, including progression to cirrhosis, development of hepatocellular carcinoma, and liver-related mortality, compared with nonsurgical management. These benefits have been observed in cohorts with biopsy-confirmed metabolic dysfunction-associated steatohepatitis (MASH) and hepatic fibrosis [6–9]. Furthermore, a dose-dependent relationship exists between the degree of surgical-induced weight loss, metabolic improvement, and histological resolution. Patients who achieve complete resolution of MASH or regression of fibrosis stage following surgery experience markedly enhanced long-term survival [10]. Beyond weight loss, emerging evidence indicates that MBS induces unique metabolic adaptations, including alterations in circulating amino acids and bile acids, modulation of the gut microbiome composition, and changes in intestinal lipid metabolism [11–15]. Importantly, several studies suggest a weight loss-independent enhancement of hepatic insulin sensitivity following surgery [16–18], implicating intrinsic hepatic mechanisms as key mediators of metabolic recovery.

The liver, as a central organ for fatty acid metabolism and energy homeostasis, is particularly vulnerable to obesity-induced metabolic stress [19]. Excessive hepatic lipid accumulation in hepatocytes, a hallmark of MASLD, reflects an imbalance between lipid uptake, de novo lipogenesis, oxidation, and export [20–22]. Sustained hepatic lipid overload underlies the progression of MASLD from simple steatosis to MASH, fibrosis, and cirrhosis [21, 22]. Because MBS reverses many of the upstream metabolic abnormalities underlying this process, it provides a unique experimental model for investigating the hepatic molecular mechanisms that mediate disease regression. Understanding how MBS reshapes these pathways is critical to understanding the biological basis of its therapeutic benefits.

In the present study, we characterize hepatic and circulatory transcriptomic changes following SG in a rat model of diet-induced obesity (DIO). By comparing liver gene expression profiles across SG-treated, DIO-obese, DIO-sham, and ad libitum-fed control animals, we aim to elucidate pathways involved in lipid metabolism and energy homeostasis, and to assess their contribution to the improvement of MASLD. Importantly, by integrating hepatic and whole-blood molecular profiling, this study identifies a previously underappreciated liver-circulatory concordance that is consistent with coordinated systemic metabolic reprogramming following DIO-SG. Because DIO-SG produced pronounced weight loss and a marked fall in hepatic triglyceride (TG), both of which independently influence metabolic gene expression, these transcriptomic changes are interpreted relative to DIO-obese and DIO-sham-operated controls rather than attributed to the surgical procedure independently of weight loss. Nevertheless, they provide insight into the molecular adaptations associated with the postoperative metabolic state and support the development of translational biomarkers and targeted strategies for obesity-related liver disease.

## Materials and Methods

### Study Design and Sample Source

This study utilized liver tissue and whole blood obtained from a previously established rat model of DIO and MASLD, subjected to SG or sham surgery, as per previous protocol in our earlier publication [23]. Briefly, male Sprague-Dawley rats (*n* = 32) were fed either a standard chow or a high-fat, high-sugar cafeteria diet for 17 weeks, followed by either SG (*n* = 8), sham surgery (*n* = 8), or no intervention (control [*n* = 8] and obese [*n* = 8] groups).

Liver tissues were collected 30 days post-surgery. Tissues were preserved in Allprotect tissue reagent (Cat No. 76405, Qiagen), or snap frozen in liquid nitrogen and stored at −80°C until further analysis. Whole blood (500 µL) was collected in RNAprotect animal tubes (Cat No. 76554, Qiagen) and stored as per the manufacturer protocol until further analysis. The remaining whole blood was centrifuged at 2,500 rpm for 10 min, and the whole blood obtained was stored in −80°C for further use. Full details of the animal model including body weight trajectories, food intake, fasting glucose, and circulating lipid profiles, together with the surgical and dietary protocols are reported in the source study [18]; in that cohort, DIO-SG produced significant and sustained weight loss and metabolic improvement relative to DIO-obese and DIO-sham-operated animals. The present study focuses on hepatic and whole-blood transcriptomic profiling and additionally reports hepatic TG content and circulating adipokine levels for the same animals.

### Ribonucleic Acid Extraction and Fatty Liver Array via Quantitative Real-Time Polymerase Chain Reaction

Total ribonucleic acid (RNA) was extracted from liver tissues and whole blood using RNA Lipid Tissue Kit (Cat. No. 74804, Qiagen) and RNeasy Protect Animal Blood Kit (Cat No. 73224, Qiagen), respectively. The extracted RNA was quantified for quality and concentration using an Epoch Microplate Spectrophotometer (BioTek Instruments, Inc.). RNA samples were reverse transcribed to produce complementary deoxyribonucleic acid using RT2 First Strand Kit (Cat. No. 330404, Qiagen). Quantitative real-time polymerase chain reaction (PCR) array was performed on QuantStudio 5 (Applied Biosystems, Inc.) using RT2 SYBR Green ROX Master Mix (Cat No. 330523, Qiagen) and RT^2^ Profiler™ PCR Array Rat Fatty Liver (Cat. No. PARN157Z-330231, Qiagen) under optimized conditions, following the manufacturer’s protocol. Relative expression was assessed using the ΔΔCt method.

### Measurement of Leptin and Adiponectin in Plasma Using Multiplex Assay

Plasma concentrations of leptin and adiponectin were measured using the MILLIPLEX^®^ MAP Rat Metabolic Adipocyte Magnetic Bead Panel (Cat. No. RMHMAG-82K, Millipore, Billerica, MA, USA). Assays were performed according to the manufacturer’s protocols.

Briefly, plasma samples were thawed on ice and centrifuged at 10,000 *g* for 10 min to remove particulates. Standards, quality controls, and plasma samples were incubated with magnetic beads coated with analyte-specific antibodies in a 96-well plate at 4°C overnight with gentle shaking. After washing to remove unbound material, detection antibodies and streptavidin-phycoerythrin conjugate were added sequentially. Plates were read using a Bio-Plex^**®**^ 200 system (Bio-Rad), and data were analyzed with xPONENT^®^ software. Analyte concentrations were calculated using standard curves generated by a five-parameter logistic regression model. Intra- and inter-assay coefficients of variation were maintained within acceptable limits (<15%).

### Biochemical Analysis of Hepatic TGs

Hepatic TG content was measured using a TG colorimetric assay kit (Cat. No. MAK266, Sigma-Aldrich) according to the manufacturer’s instructions. Approximately 100 mg of liver tissue was homogenized in 1 mL of 5% Nonidet P-40 solution using a bead mill homogenizer. The homogenates were heated to 80–100°C for 5 min to solubilize the lipids and then cooled to room temperature. This heating and cooling step was repeated twice to ensure complete solubilization. Samples were centrifuged at 12,000 *g* for 10 min, and the supernatant was collected for analysis. TG concentrations were determined by measuring absorbance at 540 nm using a microplate reader (BioTek Instruments, Inc.). Results were reported as absolute concentrations (ng/µL) based on the assay standard curve, without normalization to tissue weight, as all samples were processed using an equal tissue mass (100 mg).

### Bioinformatics and Statistical Analysis

All statistical analyses and bar graph visualizations were conducted using GraphPad Prism software version 8.0.2 (GraphPad Software, San Diego, USA). Data are presented as mean ± SEM unless otherwise specified. Statistical significance between groups was determined using one-way ANOVA followed by Tukey’s post hoc test, and *p* < 0.05 was considered statistically significant. Heatmaps and volcano plots were generated using the ggplot2 package in R (version 4.3.0) to visualize differential gene expression patterns across experimental groups. Expression data were log_2_-transformed and standardized by z-score normalization before plotting. Protein-protein interaction networks were constructed using the STRING database (version 12.0; https://string-db.org/) with a minimum required interaction score of 0.4 (medium confidence). The resulting interaction maps were analyzed to identify key hub genes and functional clusters involved in metabolic and inflammatory pathways. Functional enrichment analyses were performed using the Database for Annotation, Visualization, and Integrated Discovery (DAVID, version 2021; https://david.ncifcrf.gov/). Both Gene Ontology (GO) and Kyoto Encyclopedia of Genes and Genomes (KEGG) pathway analyses were carried out to determine significantly enriched biological processes, molecular functions, and pathways among the differentially expressed genes. Enrichment was considered statistically significant at *p* < 0.05. For circulatory biomarker analysis, data were expressed as mean ± standard error of the mean. Statistical assessment was two-sided and considered statistically significant at *p* < 0.05. Calculations were performed using GraphPad Prism, version 8.0.

## Results

### Differential Expression of Genes in Liver and Whole-Blood Cell after SG

To characterize transcriptional changes associated with metabolic improvement after SG, we analyzed the expression of key metabolic genes in liver tissue and whole blood using a targeted PCR array. Heatmap analysis of liver samples revealed a distinct clustering pattern that clearly separated DIO-obese and those undergoing SG, with DIO-SG group showing a gene expression profile resembling that of the controls (Fig. 1a). Several lipogenic and inflammatory genes were downregulated, while genes involved in fatty acid oxidation and glucose metabolism were restored toward normal levels. Several genes in liver that were elevated in DIO-obese rats showed lower expression following surgery, including *Ndufb6*, *Nr1h4*, *Dgat2*, *Srebf1*, *G6pc*, *Pparg*, *Cpt1a*, *Acadl*, *Apoa1*, and *Fabp1*. In contrast, genes such as *Ppargc1a*, *Hmgcr*, *Lpl*, *Slc2a4*, and *Ifng* were expressed at higher levels in the surgery group compared with DIO-obese animals (Fig. 1a), indicating a marked shift in hepatic gene expression after surgical intervention.

**Fig. 1.**
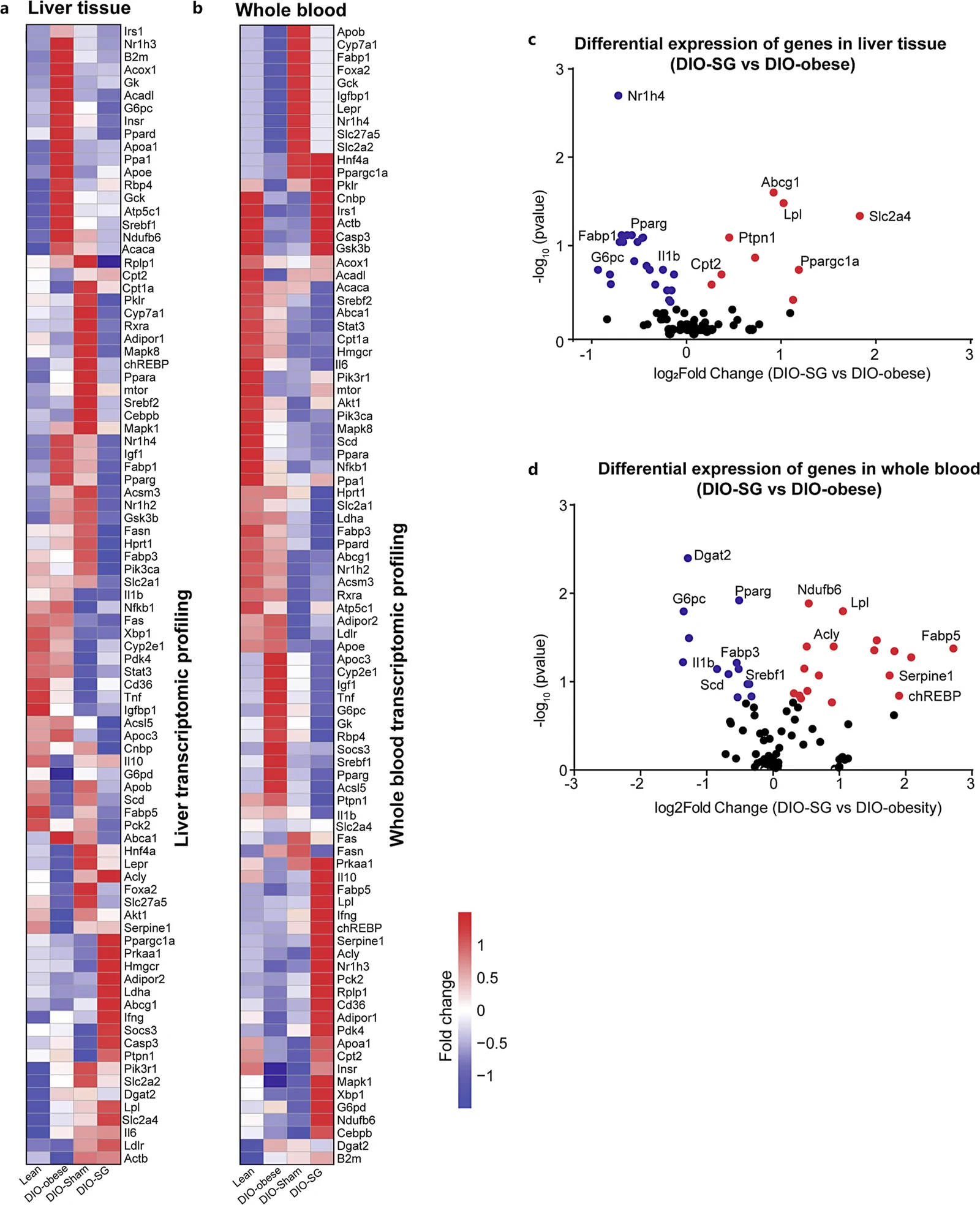
Differential metabolic gene expression in liver and whole blood assessed by RT-qPCR gene expression array. **a** Heatmap showing the relative expression (fold-change) of genes in liver tissue across lean, DIO-obese, DIO-sham, and SG groups. **b** Heatmap depicting whole blood cell gene expression changes analyzed using the same RT-qPCR gene array. Data are presented as fold-change (log_2_ scale) with hierarchical clustering. Fold-change is expressed relative to the lean control group. Red indicates upregulation, while blue denotes downregulation. **c** Volcano plots depicting differentially expressed genes in the liver comparing DIO-SG vs. DIO-obese groups. **d** Volcano plots depicting differentially expressed genes in whole blood comparing DIO-SG vs. DIO-obese groups. The x-axis shows log_2_ fold-change (obese vs. SG), and the y-axis represents −log_10_ (*p* value). Notable, significantly altered genes are labeled. Each point represents a single gene: red points denote genes significantly upregulated, and blue points genes significantly downregulated in the DIO-SG group relative to the obese (DIO-obese) group (*p* < 0.05), with nonsignificant genes shown in gray.

Similarly, the whole-blood cell gene expression profile demonstrated a comparable trend, where DIO-obese exhibited altered gene expression levels of metabolic regulators that were normalized following surgery (Fig. 1b). Genes that were increased in obesity, including *Dgat2*, *Srebf1*, *Il1β*, *Fasn*, *G6pc*, and *Pparg*, were reduced after surgery. At the same time, several genes showed higher expression in the DIO-SG group, including *Lpl*, *Insr*, *Cpt2*, *Apoa1*, *Il10*, *Irs1*, *Actb*, *Cnbp*, *Fabp5*, and *Ndufb6* (Fig. 1b), demonstrating that circulating gene expression is also strongly altered following SG. The DIO-obese and DIO-sham groups exhibited broadly similar transcriptomic profiles, with only modest differences in a limited subset of genes, consistent with a minor effect of the sham surgical procedure relative to the more pronounced changes observed following SG.

Volcano plot analysis identified key regulators distinguishing DIO-SG from DIO-obese groups (Fig. 1c, d). In the liver, *Nr1h4*, *Pparg*, *Fabp1*, and *G6pc* were strongly downregulated, while *Slc2a4*, *Lpl*, *Ptpn1*, *Abcg1*, *Ppargc1a*, and *Cpt2* were significantly upregulated (*p* < 0.05) (Fig. 1c). In whole blood, *Slc2a4*, *Dgat2*, *G6pc*, *Il1β*, *Scd*, *Fabp3*, *Pparg*, and *Srebf1* were suppressed, whereas *Ndufb6*, *Lpl*, *Fabp5*, *Serpine1*, *Mlxipl*, and *Acly* were enhanced, indicating systemic metabolic reprogramming and improved lipid handling after surgery (Fig. 1d).

### Metabolic Gene Reprogramming in the Liver after SG

To characterize hepatic transcriptional changes associated with metabolic improvement after SG, we examined the differential expression of metabolic genes in liver tissue. All data in this and the following sections are derived from the same targeted RT^2^ Profiler Fatty Liver RT-qPCR Array used above: whereas the heatmaps and volcano plots (Fig. 1) summarize array-wide expression patterns, the bar graphs presented here show RT-qPCR quantification of individual genes selected a priori for their established roles in de novo lipogenesis, gluconeogenesis, fatty acid oxidation, mitochondrial function, and inflammatory signaling, and were displayed because they were significantly differentially expressed between the DIO-SG and DIO-obese groups. Several lipogenic and energy metabolism-related genes were significantly downregulated in the DIO-SG group compared to DIO-obese group (Fig. 2a). These included *Dgat2*, *Gck*, *Srebf1*, *Fabp1*, *Pparg*, *G6pc*, *Nr1h4*, *Ndufb6*, and *Cpt1a*, which are known to promote lipid accumulation, gluconeogenesis, and oxidative stress.

**Fig. 2.**
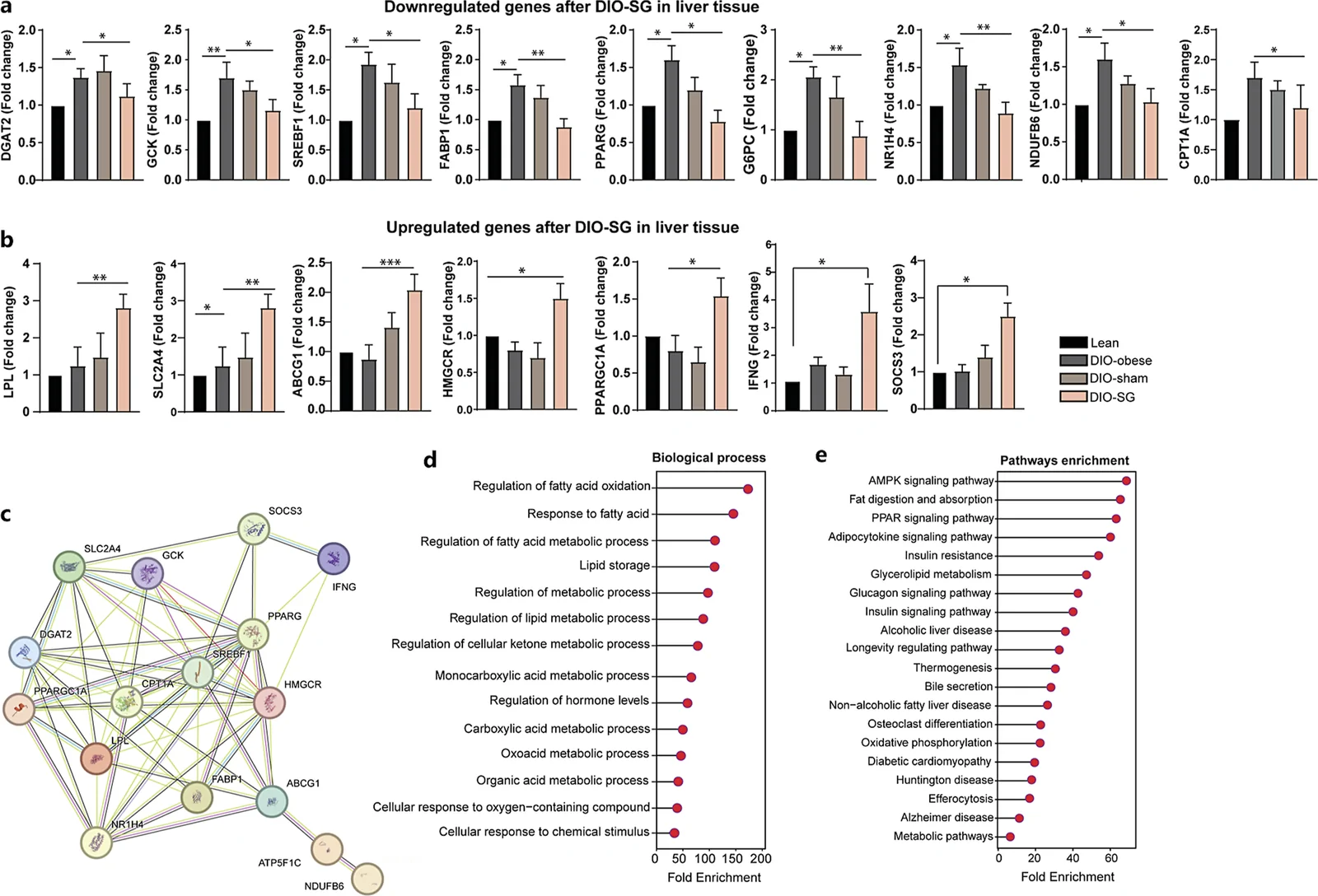
SG reverses hepatic lipogenic and metabolic dysregulation in DIO-obese rats. **a** qRT-PCR analysis of hepatic genes primarily involved in lipogenesis, glucose metabolism, and mitochondrial function across lean control, DIO-obese, DIO-sham, and DIO-SG groups. **b** Expression of genes showing significant upregulation after SG surgery relative to DIO-obese rats. **c** PPI network of differentially expressed hepatic genes illustrates strong interconnections and coordinated molecular responses induced by surgery. **d** GO enrichment analysis highlights biological processes associated with metabolic adaptation and cellular regulation after DIO-SG. **e** Functional enrichment analysis indicates overall improvement in metabolic homeostasis and energy balance in the hepatic tissue post-surgery. Data are presented as mean ± SEM (*n* = 8 per group). Statistical analysis was performed using one-way ANOVA with Tukey’s post hoc test. *p < 0.05; **p < 0.01; ***p < 0.001. Comparisons are DIO-SG versus the DIO-obese group unless otherwise indicated; the GO and KEGG analyses in **d** and **e** were performed on the whole-blood genes differentially expressed between the SG and obese groups. qRT-PCR, quantitative real-time polymerase chain reaction; PPI, protein-protein interaction.

Conversely, several metabolic regulators, including *Lpl*, *Slc2a4*, *Abcg1*, *Hmgcr*, *Ppagc1a*, *Ifng*, and *Socs3*, were significantly upregulated after surgery (Fig. 2b), reflecting improved lipid utilization, enhanced cholesterol synthesis, and improved insulin responsiveness. To examine the functional relationships among genes altered by SG, a protein-protein interaction network was constructed using the significantly differentially expressed genes identified in Figure 2a, b. The resulting network revealed a highly interconnected module centered on key regulators of hepatic lipid and glucose metabolism, indicating coordinated rather than isolated transcriptional changes. Within the network, central hub nodes included *Pparg*, *Slc2a4*, *Cpt1a*, *Dgat2*, *Fabp1*, *Hmgcr*, and *Srebf1* as key genes linking lipid and glucose metabolism (Fig. 2c). These nodes exhibited multiple high-confidence interactions, suggesting that SG modulates core metabolic control points rather than peripheral pathways. Genes involved in glucose handling and insulin sensitivity, such as *Gck* and *Slc2a4*, were closely linked to lipid metabolic regulators, highlighting crosstalk between carbohydrate and lipid metabolism in the post-surgery liver. Inflammatory and signaling-associated genes, including *Ifng* and *Socs3*, were integrated into the same network module, connecting immune signaling with metabolic regulation. This integration suggests that SG reshapes hepatic metabolism in part through modulation of inflammatory-metabolic interactions. Additional nodes related to mitochondrial function and energy metabolism (including *Ppargc1a*, *Cpt1a*) further reinforced the role of mitochondrial remodeling in the observed metabolic improvements.

Functional enrichment analyses revealed that the differentially expressed genes were predominantly associated with fatty acid oxidation, lipid storage, hormone regulation, and metabolic homeostasis (Fig. 2d). KEGG pathway analysis further emphasized activation of the adenosine monophosphate-activated protein kinase (AMPK) and proliferator-activated receptor (PPAR) signaling pathways, along with suppression of lipogenic and insulin resistance-related processes (Fig. 2e).

Taken together, these findings demonstrate that SG effectively reverses obesity-induced hepatic metabolic dysregulation by suppressing lipogenesis and enhancing lipid oxidation and glucose transport pathways, thereby restoring hepatic metabolic balance. Notably, several enrichment categories returned by the ontology analysis such as “lipid storage,” “fatty acid biosynthesis,” and “non-alcoholic fatty liver disease” group genes by biological function and are populated by transcripts regulated in both directions; their appearance therefore indicates that these pathways were coordinately remodeled after SG rather than uniformly activated. Within these categories, the core lipogenic and lipid storage drivers (*Srebf1*, *Dgat2*, *Fasn*, *Fabp1*) were downregulated, whereas genes favoring fatty acid oxidation and lipid export (*Cpt2*, *Lpl*, *Ppargc1a*) were upregulated, consistent with a net shift away from hepatic lipid storage toward oxidation.

### Whole-Blood Cell Transcriptome Shows Metabolic Recovery after SG

We evaluated circulating transcriptomic changes following SG; the expression of key metabolic and inflammatory genes was analyzed in whole-blood samples from control-lean, DIO-obese, DIO-sham, and DIO-SG groups. Several lipid synthesis and pro-inflammatory genes that were markedly elevated in the obese group, including *Dgat2*, *Il-1β*, *Srebf1*, *Fasn*, and *G6pc*, were significantly downregulated after SG (*p* < 0.05) (Fig. 3a). This reduction reflects suppression of systemic lipogenic and inflammatory pathways in response to surgical intervention. Conversely, genes associated with fatty acid oxidation and anti-inflammatory response such as *Lpl*, *Fabp5*, *Ifng*, *Acly*, *Mlxipl*, and *Il-10* were significantly upregulated in the surgery group, indicating improved lipid utilization and resolution of the obesity-induced inflammatory response (Fig. 3b).

**Fig. 3.**
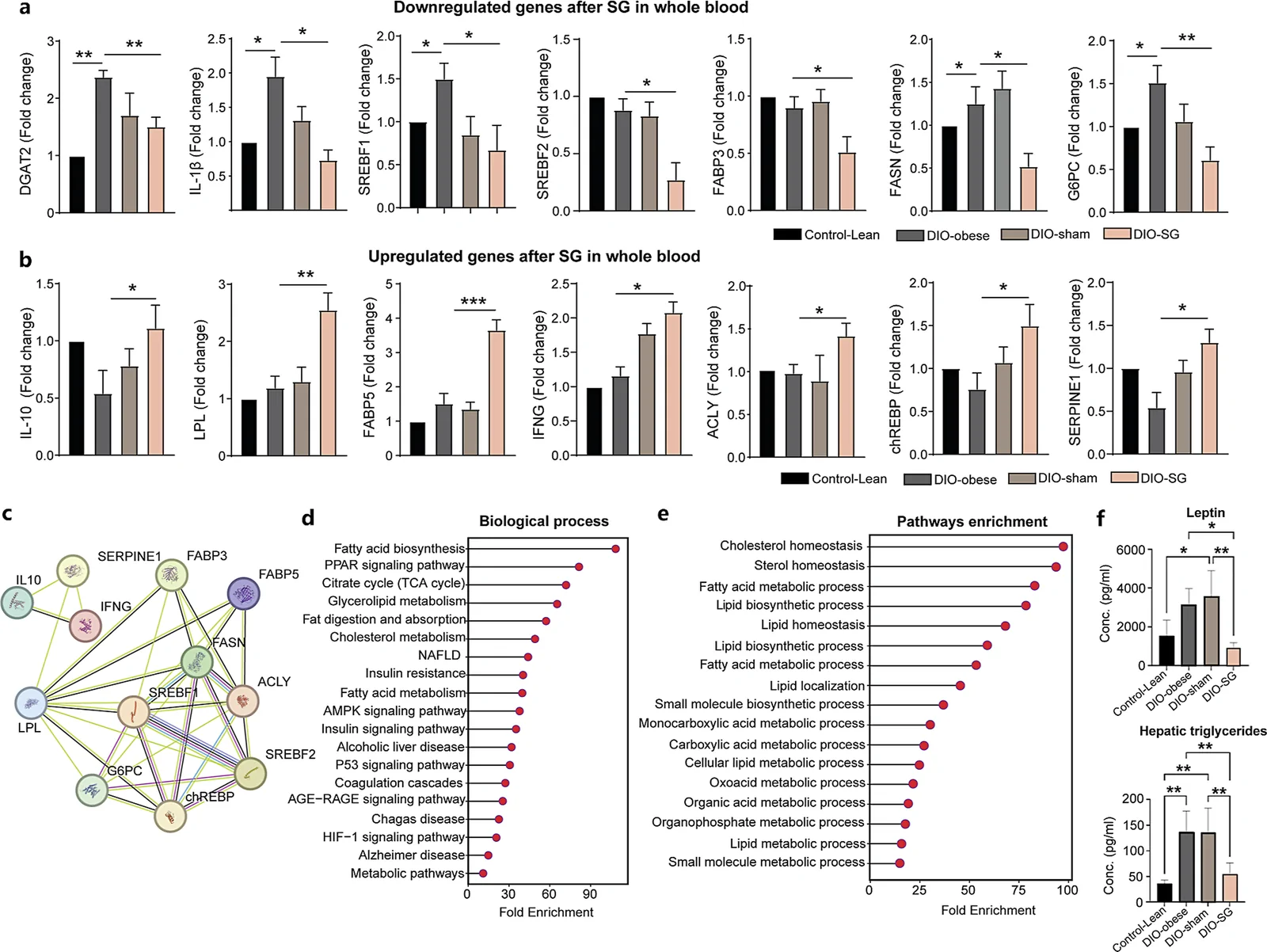
Metabolic surgery modulates circulating gene expression and metabolic pathway enrichment in obese rats. **a** qRT-PCR analysis of whole blood-derived transcripts significantly downregulated after bariatric surgery, approaching or falling below control levels. **b** Expression of whole blood genes showed significant upregulation post-surgery. **c** Protein-protein regulatory network constructed using STRING highlights interconnections among key whole blood regulators, indicating coordinated modulation of metabolic and inflammatory pathways following surgery. **d** GO enrichment analysis identifies significantly enriched biological processes associated with metabolic regulation and immune modulation. **e** KEGG enrichment analysis indicates broad transcriptional reprogramming at the systemic level after DIO-SG. **f** Circulatory levels of leptin and hepatic TG across tested groups. Data are shown as mean ± SEM (*n* = 8 per group). Statistical comparisons were performed using one-way ANOVA with Tukey’s post hoc test. *p < 0.05; **p < 0.01; ***p < 0.001. qRT-PCR, quantitative real-time polymerase chain reaction.

The network revealed extensive connectivity among genes that were significantly altered following surgery, indicating coordinated changes in gene expression rather than isolated effects. Several genes occupied central positions within the network, including *Srebf1*, *Fasn*, *Acly*, *Fabp3*, *Fabp5*, and *Lpl*, each showing multiple interactions with other differentially expressed genes (Fig. 3c). These genes were connected to both upregulated and downregulated nodes, reflecting their integration within the broader transcriptional response observed in whole blood after surgery. Inflammation-associated genes such as *Ifng* and *Il10* were also incorporated into the network and linked to metabolic genes, suggesting a close association between immune-related and metabolic gene expression changes (Fig. 3c). Additional connections were observed among genes involved in lipid handling and transcriptional regulation, including *Mlxipl*, *Serpine1*, and *Srebf2*.

Functional enrichment analysis revealed distinct pathway activations following SG. GO analysis identified significant enrichment in biological processes related to fatty acid biosynthesis and PPAR signaling, indicating improved regulation of lipid metabolism (Fig. 3d). Similarly, KEGG pathway analysis demonstrated activation of AMPK and cholesterol metabolism pathways, reflecting a coordinated shift toward enhanced lipid utilization and restored energy balance after SG (Fig. 3e). As in the hepatic analysis, enrichment of terms such as “fatty acid biosynthesis” and “non-alcoholic fatty liver disease” reflects the functional annotation of the regulated gene set rather than net upregulation of lipogenesis: the individual lipogenic transcripts within these terms (*Srebf1*, *Fasn*, *Dgat2*) were in fact downregulated after SG, whereas oxidative and lipid-handling genes (*Lpl*, *Fabp5*) were upregulated.

### Plasma Hormone Profiles of Leptin, Adiponectin, and Hepatic TGs following SG

Plasma concentrations of leptin differed significantly across the study groups, whereas adiponectin levels remained unchanged. Leptin concentrations were substantially elevated in both the DIO-obese (3,121.0 ± 491.5 pg/mL) and DIO-sham (3,575.0 ± 590.3 pg/mL, *p* ≤ 0.02) compared to the control-lean animals (1,542 ± 360.0 pg/mL). SG resulted in marked reduction in leptin levels in DIO-SG group (937.8 ± 122.6 pg/mL), significantly lower than both DIO-obese (*p* ≤ 0.04) and DIO-sham (*p* ≤ 0.005) groups, with values approximating those observed in lean controls (Fig. 3f). Hepatic TG content was significantly elevated in the DIO-obese (137.1 ± 16.5 ng/μL, *p* ≤ 0.002) and DIO-sham (135.3 ± 19.5 ng/μL, *p* ≤ 0.003) groups compared to the control-lean group (36.6 ± 3.4). Importantly, SG markedly reduced hepatic TG levels in the DIO-SG group (54.5 ± 9.8 ng/µL), with values approaching those of lean controls and significantly lower than both the DIO-obese (*p* ≤ 0.007) and DIO-sham (*p* ≤ 0.008) groups (Fig. 3f).

### MBS Reprograms Liver and Whole-Blood Metabolic Crosstalk

Integrated analysis of liver and whole-blood transcriptomic data revealed shared gene expression changes that reflect coordinated systemic reprogramming following SG (Fig. 4). Pathway mapping showed that lipogenic genes, *Dgat2*, *Acaca*, *Fasn*, *Scd*, *Mlxipl*, *Srebf1*, and *Srebf2*, which drive fatty acid and TG synthesis, were downregulated post-surgery. Here, we observed that genes promoting fatty acid oxidation and energy utilization, including *Lpl*, *Cpt2*, *Fabp1* and *Fabp5*, were upregulated. Notably, *G6pc*, a key gluconeogenic gene, was inversely associated with *Mlxipl* downregulation, suggesting a metabolic shift from glucose production toward enhanced glucose utilization and storage. Upregulation of *Slc2a4* and *Hmgcr* further supported improved glucose uptake and cholesterol homeostasis. Additionally, increased *Il10*, *Serpine1*, and decreased *Il1β* expression indicated attenuation of inflammation regulation. Therefore, this study highlights a coordinated hepatic-whole-blood regulatory axis that balances glucose, lipid, and inflammatory pathways after SG.

**Fig. 4.**
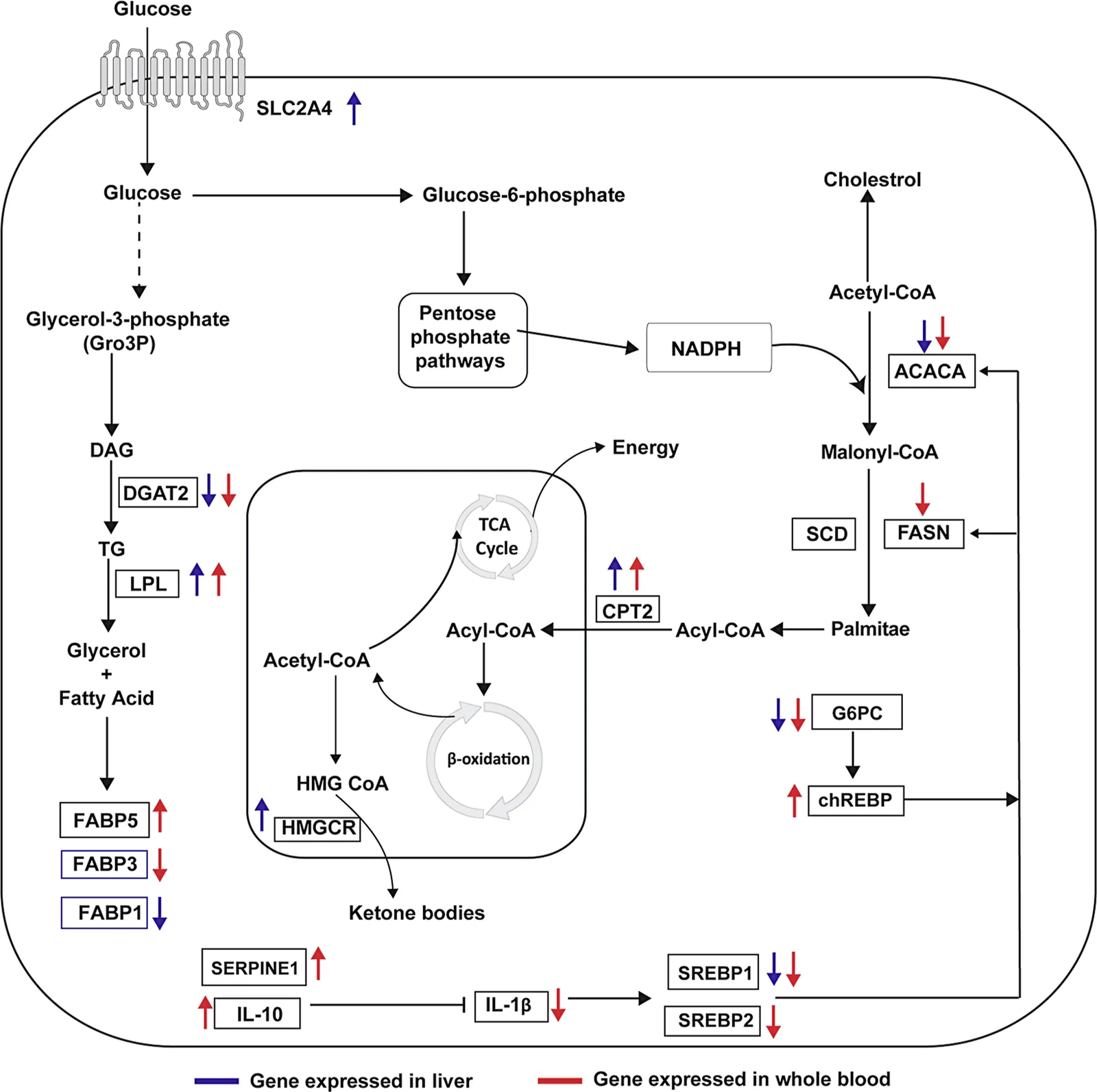
Integrated analysis of liver and whole blood metabolic gene expression and pathway modulation following SG surgery. Schematic representation of key metabolic pathways illustrating the expression changes of overlapping genes in lipid and glucose metabolism after bariatric surgery. Red arrows indicate genes upregulated after surgery. Blue arrows indicate genes downregulated after surgery. Black arrows represent metabolic flux directions within pathways. The figure is an interpretive schematic of a representative hepatocyte and does not depict a specific cell imaged in this study; it integrates the concordant hepatic and whole-blood expression changes observed here and was prepared using Adobe Illustrator and Bioicons (https://bioicons.com/). It summarizes, rather than statistically infers, the liver-circulation relationships and should be interpreted accordingly.

## Discussion

In this study, SG markedly attenuated high-fat diet-induced hepatic steatosis in obese rats by reversing lipogenic and gluconeogenic gene programs while enhancing fatty acid oxidation and mitochondrial pathways. These molecular changes were accompanied by significant reductions in circulating leptin, together with a marked fall in hepatic TG content, reflecting systemic metabolic improvement. Pathway analysis revealed normalization of PPAR, AMPK, and inflammatory pathways signaling with concomitant improvement in insulin sensitivity. Notably, the strong concordance between hepatic and whole-blood molecular signatures represents a key novel finding of this study, highlighting SG-induced systemic metabolic reprogramming that extends beyond weight loss alone and supports the translational potential of circulating biomarkers for monitoring hepatic response to metabolic surgery.

This study provides molecular and biochemical evidence that SG reverses obesity-induced hepatic and systemic metabolic dysregulation through coordinated transcriptional reprogramming of lipid metabolism, mitochondrial activity, and inflammatory signaling. Downregulation of lipogenic genes (*Srebf1*, *Dgat2*, *Fasn*) and upregulation of oxidative regulators (*Cpt2*, *Ppargc1a*) indicate a metabolic shift toward enhanced lipid oxidation and energy homeostasis, accompanied by reduced hepatic fat accumulation. The hepatic changes were associated with improvements in circulating biomarkers, including reductions in circulating leptin and in hepatic TG, supporting a recovery of systemic metabolic balance. In our earlier work [23], we demonstrated that SG alters the circulating levels of key gut hormones, particularly increasing circulating glucagon-like peptide-1, gastric inhibitory polypeptide, and glucagon post-surgery. Taken together, the concordant modulation of hepatic and whole-blood gene networks highlights dynamic liver-circulation crosstalk, reinforcing the systemic nature of metabolic improvement following SG, reinforcing systemic benefits that extend beyond local hepatic effects.

As expected, obesity was associated with the upregulation of lipogenic and inflammatory genes including *Fasn*, *Acly*, *Srebf1*, *Nfkb1*, and *Il1b*, consistent with enhanced lipid synthesis, hepatic steatosis, and inflammation [24–27]. Following SG, these genes were significantly downregulated, indicating suppression of de novo lipogenesis and inflammatory signaling. Normalization of *Srebf1* and *Dgat2* expression in particular [28, 29] key regulators of cholesterol, fatty acid, and TG synthesis highlights a molecular shift toward restored hepatic lipid homeostasis and improved insulin sensitivity.

Concurrently, SG induced marked upregulation of genes involved in mitochondrial β-oxidation, cholesterol efflux, and energy metabolism, including *Ppargc1a*, *Cpt2*, *Lpl*, and *Abcg1*, alongside increased expression of anti-inflammatory mediators such as *Ifng* and *Il10*. These transcriptional changes collectively suggest enhanced oxidative metabolism and resolution of metabolic inflammation. GO and KEGG pathway analyses supported these observations, revealing enrichment in fatty acid oxidation, PPAR signaling, AMPK activation, and insulin sensitivity pathways, consistent with restoration of hepatic metabolic flexibility and energy balance.

A key observation of this study is the strong concordance between hepatic and circulating gene expression patterns following surgery. Downregulation of *Fasn*, *Srebf1*, and *Dgat2*, together with the upregulation of *Lpl*, *Il10*, and *Cpt2* in whole blood, mirrors hepatic trends and suggests that circulating transcripts can reflect intrahepatic metabolic status. This systemic concordance supports the concept of cross-tissue metabolic coordination and provides the basis for developing circulating biomarkers to monitor metabolic recovery after SG. Importantly, these findings provide a rationale for investigating circulating transcriptomic signatures as minimally invasive surrogate markers of hepatic metabolic remodeling after bariatric surgery, although validation in independent cohorts and human studies will be required before clinical application.

Network analyses identified *Ppargc1a*, *Srebf1*, *Cpt2*, *Dgat2*, and *Lpl* as key regulatory hubs that integrate lipid synthesis, fatty acid oxidation, and inflammatory signaling, underscoring their pivotal role in the metabolic reprogramming induced by SG. Upregulation in the gene expression of *Ppargc1a* and *Cpt2* may reflect enhanced mitochondrial biogenesis and β-oxidation capacity, which would align with improved hepatic energy metabolism and reduced steatosis. In contrast, the downregulation of lipogenic drivers as seen in the genetic expression of *Srebf1* and *Dgat2* may indicate suppression of de novo lipogenesis and TG synthesis, which would align with reduced hepatic lipid accumulation observed after surgery. The increased expression of *Lpl*, a key regulator at the interface of lipid transport and clearance, contributes to enhanced whole-blood lipid turnover and promotes the redistribution of fatty acids toward oxidative pathways rather than storage. Collectively, these transcriptional shifts culminate on the activation of PPAR and AMPK signaling pathways, alongside the attenuation of inflammatory mediators such as *IL1β* and *Serpine1*. These coordinated molecular adaptations implicate the restoration of systemic lipid and glucose homeostasis beyond the effects of weight loss alone, aligning with results from a prior study demonstrating weight-loss-independent improvements in hepatic insulin sensitivity after surgery [16] as well as multiomic analyses of steatotic livers following dietary restriction and SG [30]. However, because the present study did not include a weight-matched control group, it is not possible to determine from our data whether these transcriptional changes are truly independent of weight loss and the accompanying reduction in hepatic steatosis.

Several limitations should be acknowledged. The study employed a targeted transcriptional profiling array rather than an unbiased next-generation sequencing approach, which may have limited the identification of novel pathways. An important limitation of this study is the lack of a weight-matched (calorie-restricted or pair-fed) control group. Because SG produced marked weight loss and a substantial reduction in hepatic TG content, both recognized drivers of metabolic and inflammatory transcriptional remodeling. The observed transcriptomic changes cannot be unequivocally attributed to the surgical intervention independent of weight loss. Instead, these findings should be interpreted as molecular adaptations associated with the overall metabolic improvements following SG, relative to obesity and sham-operated controls. Future studies incorporating weight-matched controls will be important to distinguish surgery-specific effects from those driven primarily by weight loss. A further limitation is the use of whole blood, which comprises multiple leukocyte populations; consequently, apparent changes in bulk gene expression may partly reflect shifts in cell-type composition rather than altered per-cell transcription. Future studies employing single-cell RNA sequencing, sorted immune cell populations, or computational cell deconvolution approaches will be valuable for distinguishing changes in cellular composition from true transcriptional regulation within specific blood cell populations. In addition, the integrative liver-blood pathway model shown in Figure 4 is interpretive and derived from the observed expression changes rather than from formal cross-tissue statistical inference; corroboration with independent, publicly available transcriptomic datasets is warranted. Furthermore, gene expression changes were not validated at the protein or functional level using complementary methodologies, such as immunohistochemistry or proteomic analyses. Finally, the relatively short post-surgical follow-up and use of a single experimental model may limit assessment of long-term hepatic remodeling and broader translational applicability.

## Conclusion

This study demonstrates that SG exerts systemic metabolic benefits through dual-tissue transcriptional reprogramming of lipid, energy, and inflammatory pathways. The parallel regulation observed in both liver and whole blood highlights a coordinated response that may serve as a molecular basis for the clinical efficacy of bariatric procedures. Furthermore, the identification of overlapping hepatic-whole-blood gene networks provides opportunities for the development of minimally invasive biomarkers to monitor metabolic recovery in obesity-related diseases after MBS. Future studies should validate these candidate genes in human cohorts and explore their predictive utility for long-term remission of obesity-associated comorbidities, including type 2 diabetes and MASLD.

## Acknowledgments

The investigators sincerely thank the Kuwait Foundation for the Advancement of Sciences (KFAS) for providing financial support for this research (Project No. CN19-13MM-01). The investigators also thank the Director and staff of the Research Core Facility (SRUL02/13 and GM01/15), Health Sciences Center, Kuwait University, for providing the facilities and technical support necessary for this research.

## Statement of Ethics

The study was approved by the Institutional Ethics Committee of the Research, College of Medicine, Kuwait University (VDR/EC/3689) and all experimental procedures complied with institutional and international standards.

## Conflict of Interest Statement

Dr. Suleiman Al-Sabah is an Editorial Board Member of Medical Principles and Practice. All other authors declare that they have no conflicts of interest.

## Funding Sources

The study was supported by the Kuwait Foundation for the Advancement of Sciences (Project No. CN19-13MM-01).

## Author Contributions

Conceptualization: Mohammad Jamal. Methodology: Mohammad Jamal, Suleiman Al-Sabah, and Carol Dsouza. Data collection: Mohammad Jamal, Carol Dsouza, and Fatemah AlOtaibi. Formal analysis: Wayel Jasem, Mohamed Abu-Farha, Fahd Al-Mulla, and Jehad Abubaker. Investigation: Mohammad Jamal, Wayel Jasem, and Jehad Abubaker. Resources: Mohammad Jamal, Carol Dsouza, and Suleiman Al-Sabah. Data curation: Mohammad Jamal, Wayel Jasem, Mohamed Abu-Farha, Carol Dsouza, Fatemah AlOtaibi, Fahd Al-Mulla, and Jehad Abubaker. Writing – original draft preparation: Mohammad Jamal, Wayel Jasem, Mohamed Abu-Farha, Suleiman Al-Sabah, Carol Dsouza, Hamad Ali, and Jehad Abubaker. Writing – review and editing: all authors. Project administration: Mohammad Jamal, Wayel Jasem, Carol Dsouza, and Yun Ma. Funding acquisition: Mohammad Jamal and Wayel Jasem. All authors have read and approved the final manuscript.

## Data Availability

The data supporting the findings of this study are available through the corresponding authors upon request.

## References

[1] Srivastava G , Apovian CM. Current pharmacotherapy for obesity. Nat Rev Endocrinol. 2018;14(1):12–24. 10.1038/nrendo.2017.122 29027993

[2] The GBD 2015 Obesity Collaborators . Health effects of overweight and obesity in 195 countries over 25 years. N Engl J Med. 2017;377(1):13–27. 10.1056/NEJMoa1614362 PMC5477817 28604169

[3] Bray GA , Heisel WE, Afshin A, Jensen MD, Dietz WH, Long M, . The science of obesity management: an endocrine society scientific statement. Endocr Rev. 2018;39(2):79–132. 10.1210/er.2017-00253 PMC5888222 29518206

[4] Yang Z , Liu Z, Liu W, Dong XC. Steatotic liver disease and cancer: from pathogenesis to therapeutic targets. eGastroenterology. 2025;3:e100218. 10.1136/egastro-2025-100218 PMC12519389 41098801

[5] Poljo A , Kraljević M, Peterli R, Müller BP, Billeter AT. Role of sleeve gastrectomy in improving metabolic syndrome: an overview. Updates Surg. 2025;77(7):2031–6. 10.1007/s13304-024-02038-4 PMC12540589 39586962

[6] Tilg H , Petta S, Stefan N, Targher G. Metabolic dysfunction–associated steatotic liver disease in adults: a review. JAMA. 2026;335(2):163–74. 10.1001/jama.2025.19615 41212550

[7] Cusi K , Abdelmalek MF, Apovian CM, Balapattabi K, Bannuru RR, Barb D, . Metabolic Dysfunction–Associated Steatotic Liver Disease (MASLD) in people with diabetes: the need for screening and early intervention. A consensus report of the American diabetes association. Diabetes Care. 2025;48(7):1057–82. 10.2337/dci24-0094 40434108

[8] Cusi K , Isaacs S, Barb D, Basu R, Caprio S, Garvey WT, . American association of clinical endocrinology clinical practice guideline for the diagnosis and management of nonalcoholic fatty liver disease in primary care and endocrinology clinical settings. Endocr Pract. 2022;28(5):528–62. 10.1016/j.eprac.2022.03.010 35569886

[9] Aminian A , Al-Kurd A, Wilson R, Bena J, Fayazzadeh H, Singh T, . Association of bariatric surgery with major adverse liver and cardiovascular outcomes in patients with biopsy-proven nonalcoholic steatohepatitis. JAMA. 2021;326(20):2031–42. 10.1001/jama.2021.19569 PMC8587225 34762106

[10] Lassailly G , Caiazzo R, Goemans A, Chetboun M, Gnemmi V, Labreuche J, . Resolution of metabolic dysfunction-associated steatohepatitis with no worsening of fibrosis after bariatric surgery improves 15-year survival: a prospective cohort study. Clin Gastroenterol Hepatol. 2025;23(9):1567–76.e9. 10.1016/j.cgh.2024.10.025 39709138

[11] Laferrère B , Reilly D, Arias S, Swerdlow N, Gorroochurn P, Bawa B, . Differential metabolic impact of gastric bypass surgery versus dietary intervention in Obese diabetic subjects despite identical weight loss. Sci Transl Med. 2011;3(80):80re2. 10.1126/scitranslmed.3002043 PMC3656497 21525399

[12] Patti M , Houten SM, Bianco AC, Bernier R, Larsen PR, Holst JJ, . Serum bile acids are higher in humans with prior gastric bypass: potential contribution to improved glucose and lipid metabolism. Obesity. 2009;17(9):1671–7. 10.1038/oby.2009.102 PMC4683159 19360006

[13] Furet JP , Kong LC, Tap J, Poitou C, Basdevant A, Bouillot JL, . Differential adaptation of human Gut microbiota to bariatric surgery–induced weight loss. Diabetes. 2010;59(12):3049–57. 10.2337/db10-0253 PMC2992765 20876719

[14] Stefater MA , Sandoval DA, Chambers AP, Wilson–Pérez HE, Hofmann SM, Jandacek R, . Sleeve gastrectomy in rats improves postprandial lipid clearance by reducing intestinal triglyceride secretion. Gastroenterology. 2011;141(3):939–49.e9494. 10.1053/j.gastro.2011.05.008 PMC3163733 21699773

[15] Liu H , Lefere S, Guillot A, Zheng MH, Tacke F. Bariatric surgery for metabolic dysfunction-associated steatotic liver disease (MASLD): current knowledge of mechanisms. Hepatology. 2025. 10.1097/HEP.0000000000001417 40445858

[16] Abu-Gazala S , Horwitz E, Ben-Haroush Schyr R, Bardugo A, Israeli H, Hija A, . Sleeve gastrectomy improves glycemia independent of weight loss by restoring hepatic insulin sensitivity. Diabetes. 2018;67(6):1079–85. 10.2337/db17-1028 PMC5961409 29475831

[17] Ben-Haroush Schyr R , Al-Kurd A, Moalem B, Permyakova A, Israeli H, Bardugo A, . Sleeve gastrectomy suppresses hepatic glucose production and increases hepatic insulin clearance independent of weight loss. Diabetes. 2021;70(10):2289–98. 10.2337/db21-0251 PMC8576500 34341005

[18] Chambers AP , Jessen L, Ryan KK, Sisley S, Wilson–Pérez HE, Stefater MA, . Weight-independent changes in blood glucose homeostasis after gastric bypass or vertical sleeve gastrectomy in rats. Gastroenterology. 2011;141(3):950–8. 10.1053/j.gastro.2011.05.050 PMC3163814 21699789

[19] Guo Y , Luan H, Lin J. Obesity is the culprit behind fatty acid-induced inflammation. Nutr Metab. 2026;23(1):35. 10.1186/s12986-026-01076-6 PMC12983907 41721408

[20] Alves-Bezerra M , Cohen DE. Triglyceride metabolism in the liver. Compr Physiol. 2017;8:1–8. 10.1002/cphy.c170012 PMC6376873 29357123

[21] Friedman SL , Neuschwander-Tetri BA, Rinella M, Sanyal AJ. Mechanisms of NAFLD development and therapeutic strategies. Nat Med. 2018;24(7):908–22. 10.1038/s41591-018-0104-9 PMC6553468 29967350

[22] Lu Q , Tian X, Wu H, Huang J, Li M, Mei Z, . Metabolic changes of hepatocytes in NAFLD. Front Physiol. 2021;12:710420. 10.3389/fphys.2021.710420 PMC8437340 34526911

[23] Al-Sabah S , Jamal MH, Al-Khaledi G, Dsouza C, AlOtaibi F, Al-Ali W, . Increased glucagon immunoreactivity in a rat model of diet-induced obesity following sleeve gastrectomy. Med Princ Pract. 2023;32(4–5):232–41. 10.1159/000533746 PMC10659591 37634505

[24] McGlone ER , Siebert M, Dore M, Hope DCD, Davies I, Owen B, . Sleeve gastrectomy causes weight-loss independent improvements in hepatic steatosis. Liver Int. 2023;43(9):1890–900. 10.1111/liv.15614 PMC10947097 37208943

[25] Xu W , Hu X, Anwaier A, Wang J, Liu W, Tian X, . Fatty acid synthase correlates with prognosis-related abdominal adipose distribution and metabolic disorders of clear cell renal cell carcinoma. Front Mol Biosci. 2021;7:610229. 10.3389/fmolb.2020.610229 PMC7868388 33569391

[26] Eberlé D , Hegarty B, Bossard P, Ferré P, Foufelle F. SREBP transcription factors: master regulators of lipid homeostasis. Biochimie. 2004;86(11):839–48. 10.1016/j.biochi.2004.09.018 15589694

[27] Greten FR , Arkan MC, Bollrath J, Hsu LC, Goode J, Miething C, . NF-κB is a negative regulator of IL-1β secretion as revealed by genetic and pharmacological inhibition of IKKβ. Cell. 2007;130(5):918–31. 10.1016/j.cell.2007.07.009 PMC2134986 17803913

[28] Amin NB , Saxena AR, Somayaji V, Dullea R. Inhibition of diacylglycerol acyltransferase 2 versus diacylglycerol acyltransferase 1: potential therapeutic implications of pharmacology. Clin Ther. 2023;45(1):55–70. 10.1016/j.clinthera.2022.12.008 36690550

[29] Yenilmez B , Wetoska N, Kelly M, Echeverria D, Min K, Lifshitz L, . An RNAi therapeutic targeting hepatic Dgat2 in a genetically obese mouse model of nonalcoholic steatohepatitis. Mol Ther. 2022;30(3):1329–42. 10.1016/j.ymthe.2021.11.007 PMC8899521 34774753

[30] Chen S , Zeng Q, Cai X, Xue J, Yin G, Song P, . Multiomics analyses decipher intricate changes in the cellular and metabolic landscape of steatotic livers upon dietary restriction and sleeve gastrectomy. Int J Biol Sci. 2024;20(11):4438–57. 10.7150/ijbs.98362 PMC11380448 39247824

